# Feasibility of Spin‐ and Gradient‐Echo Dynamic Susceptibility Contrast MRI for Microvascular Characterization at 7 Tesla

**DOI:** 10.1002/nbm.70380

**Published:** 2026-09-02

**Authors:** Karen N. van der Werff, Daniëlle van Dorth, Krishnapriya Venugopal, Esther A. H. Warnert, Dirk H. J. Poot, Frans M. Vos, Marion Smits, Stefan Klein, C. Chad Quarles, Juan A. Hernandez‐Tamames, Johan A. F. Koekkoek, Jeroen de Bresser, Matthias J. P. van Osch

**Affiliations:** ^1^ Department of Radiology and Nuclear Medicine Erasmus MC, University Medical Center Rotterdam Rotterdam the Netherlands; ^2^ TU Delft Department of Imaging Physics Delft the Netherlands; ^3^ Medical Delta Delft the Netherlands; ^4^ C. J. Gorter MRI Center, Department of Radiology Leiden University Medical Center Leiden the Netherlands; ^5^ Brain Tumor Center Erasmus MC Cancer Institute Rotterdam the Netherlands; ^6^ Department of Cancer Systems Imaging University of Texas MD Anderson Cancer Center Houston Texas USA; ^7^ Department of Neurology Leiden University Medical Center Leiden the Netherlands; ^8^ Department of Radiology Leiden University Medical Center Leiden the Netherlands

**Keywords:** 7 Tesla (7 T), dynamic susceptibility contrast (DSC), glioma, perfusion, spin‐ and gradient‐echo (SAGE), vascular architectural imaging (VAI), vessel size imaging (VSI)

## Abstract

Perfusion MRI, especially dynamic susceptibility contrast (DSC), is vital for glioma diagnosis and monitoring. While gradient‐echo DSC is the current recommended implementation, combined spin‐ and gradient‐echo (SAGE) DSC additionally allows for vessel size imaging (VSI) and has shown feasibility for microvascular characterization at 3 T. Higher field strength MRI offers increased contrast, resolution and signal‐to‐noise ratio, providing potential to improve microvascular characterization.

This feasibility study aimed to implement a protocol for SAGE‐DSC at 7 T and study its potential for microvascular characterization. SAGE‐DSC was acquired in eight patients undergoing treatment for glioma (four females, median age 57 years) at 3 and 7 T. Data were analyzed using the temporal signal‐to‐noise ratio (tSNR) and contrast‐to‐noise ratio (CNR) and by comparing perfusion maps, VSI and vascular architectural imaging (VAI). Simulations were performed to study the relationship between vessel size and $∆R_{2}^{\left(*\right)}$ for SAGE‐DSC at 3 and 7 T.

Contrast agent dosage could be reduced while maintaining higher CNR at 7 T (but lower tSNR). Assessment by an experienced neuroradiologist showed similar rCBV map quality at 3 and 7 T. VSI maps showed the same normal‐appearing gray‐to‐white matter ratios while in‐plane resolution improved at least 52% at 7 T. VAI hysteresis loops at both field strengths traversed the same counter‐clockwise direction, with increased loop areas reflecting increased susceptibility effects at 7 T. Simulations confirmed the higher sensitivity of gradient‐echo and the higher specificity of spin‐echo to the microvasculature, with peak spin‐echo specificity moving from 3 μm at 3 T towards 1.5 μm at 7 T.

This study confirmed the feasibility of using SAGE‐DSC at 7 T for microvascular characterization. rCBV and VSI maps show similar behavior while allowing for reduced contrast agent dosage and improved resolution. The spin‐echo specificity shifted towards smaller vessel radii for increasing field strengths. These findings form the basis for exploration and validation in larger patient populations.

## Graphical abstract

Spin‐ and‐gradient‐echo DSC MRI at 7 T allows for characterization of the microvasculature at an improved resolution and a reduced gadolinium‐based contrast agent dosage while maintaining increased vascular contrast. Ratios between normal‐appearing gray matter and white matter for relative cerebral blood volume and vessel size estimates were similar between 3 and 7 T. Sensitivity of the gradient‐echo and specificity of the spin‐echo shift towards smaller vessel radii at 7 T.

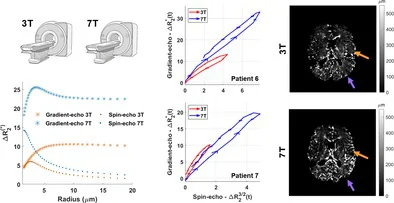

**Abbreviations**
BSW: Boxerman‐Schmainda‐Weisskoff (leakage correction method)
CNR: contrast‐to‐noise ratio
DSC‐HEPI: dynamic susceptibility contrast hybrid EPI (other name for SAGE‐DSC, specific to GE Healthcare)
GBCA: gadolinium‐based contrast agent
IDH: isocitrate dehydrogenase
MGMT: methylguanine methyltransferase
NAGM: normal‐appearing gray matter
NAWM: normal‐appearing white matter
rCBV: relative cerebral blood volume
SAGE‐DSC: spin‐ and gradient‐echo dynamic susceptibility contrast MRI
TMZ: temozolomide
tSNR: temporal signal‐to‐noise ratio
VAI: vascular architectural imaging
VSI: vessel size imaging
WHO: World Health Organization

## 1 Introduction

Dynamic susceptibility contrast (DSC) MRI is a valuable tool for diagnosing and monitoring patients with glioma. Following the intravenous injection of a gadolinium‐based contrast agent (GBCA), the shortening of T_1_, T_2_ and T_2_* relaxation times leads to measurable changes in the MR signal that can be exploited for perfusion imaging. As these changes are also sensitive to the vascular structure, they can be used to characterize the tumor's vasculature [1]. Because vascular and hemodynamic features correlate to glioma subtype and grade, DSC‐MRI provides important information for diagnosis (e.g., predicting grade and survival) and during follow‐up (e.g., distinguishing treatment‐related effects from true progression) [2, 3, 4, 5, 6].

Although gradient‐echo DSC is the recommended clinical standard [6], the use of a combined spin‐ and gradient‐echo (SAGE) technique can provide useful additional information. The gradient‐echo (GRE) sequence has a high sensitivity to a broad range of vessel sizes, whereas the spin‐echo (SE) has greater microvascular specificity [7]. By exploiting these differences, information on the vessel size can be obtained [8]. Kiselev et al. [9] validated vessel size imaging (VSI) in humans and Chakhoyan et al. [10] showed that VSI accurately reflects vessel caliber in high‐grade gliomas. Moreover, Arzanforoosh et al. [11] demonstrated the ability of VSI to distinguish glioblastoma from less aggressive glioma types, even when contrast‐enhancement is lacking. Vascular architectural imaging (VAI) exploits the temporal shift between the SE‐ and GRE‐DSC signals to produce hysteresis loops indicative of vessel caliber and oxygenation [4, 9]. This technique proved useful in identifying patients who might benefit from antiangiogenic therapy and in helping to distinguish tumor progression from pseudo‐progression [12].

Despite the clinical feasibility of SAGE‐DSC at 3 T and the theoretical advantages of higher field strengths, SAGE‐DSC has not yet been studied in humans at 7 T. Higher field strengths can provide improved signal‐to‐noise ratio (SNR), contrast‐to‐noise ratio (CNR) or spatial resolution, while the increased susceptibility effects allow for reduced GBCA doses [13]. A few studies have explored the use of GRE‐DSC at 7 T [13, 14, 15, 16] and shown that reliable perfusion measures can be obtained at a reduced GBCA dose. Next to that, Elschot et al. [17] used SE‐DSC at 7 T to image the microvasculature in older adults, confirming its applicability for measuring cerebral perfusion. The increased CNR at 7 T could encourage the clinical use of SE‐DSC, which has thus far been limited because of its poor CNR at lower field strengths. The addition of a GRE readout within the same repetition time would increase the overall vascular sensitivity and allow for high‐resolution VSI and VAI.

Accordingly, this feasibility study aims to (a) implement a protocol for SAGE‐DSC at 7 T and (b) study its potential for microvascular characterization of glioma in adult patients. To this end, rCBV maps, VSI maps and VAI loops will be compared between 3 and 7 T and gauged against signal behavior simulations.

## 2 Methods

### 2.1 Patient Characteristics

This prospective study was performed as part of the IRB‐approved vascular signature mapping (VSM) study (ClinicalTrials.gov ID: NCT05274919). All patients provided written informed consent. Patients under treatment for adult‐type diffuse glioma and scheduled for a routine follow‐up MRI in Erasmus MC (EMC) or Leiden University Medical Center (LUMC) were recruited. Out of 16 patients in this cohort, eight patients consented to an additional 7 T MRI scan at LUMC and were accordingly included in this analysis. Characteristics of the patient group are described in Table 1.

**TABLE 1:** Patient characteristics including tumor diagnosis and treatment history. The seventh column denotes the time between the last therapy and the first scan from our study, rounded to the nearest month.

#	Age (years)	Sex	Weight (kg)	Tumor diagnosis	Treatment history	Time between therapy and scan	Hospital where 3 T scan was performed
1	80	F	84	Glioblastoma, IDH‐wildtype, grade 4, MGMT‐methylated	Resection, chemoradiotherapy with Perry scheme	9 months	LUMC
2	61	M	59	Glioblastoma, IDH‐wildtype, grade 4, MGMT‐unmethylated	Resection, short radiotherapy scheme without TMZ	3 months	LUMC
3	37	M	100	Astrocytoma, IDH‐mutant, grade 2	Resection	2 years and 8 months	EMC
4	49	F	81	Astrocytoma, IDH‐mutant, grade 2	Resection, proton therapy, adjuvant TMZ	2 years and 2 months	LUMC
5	73	M	74	Glioblastoma, IDH‐wildtype, grade 4, MGMT‐unmethylated	Biopsy, chemoradiotherapy with Stupp scheme	3 years	LUMC
6	52	F	66	Oligodendroglioma, IDH‐mutant, 1p/19q‐codeleted, grade 2	Resection, chemotherapy (lomustine, followed by TMZ)	3 years and 4 months	EMC
7	66	F	71	Glioblastoma, IDH‐wildtype, grade 4, MGMT‐methylated	Resection and reresection, chemoradiotherapy with Stupp scheme, adjuvant TMZ	1 year	LUMC
8	29	M	85	Astrocytoma, IDH‐mutant, grade 3	Resection, proton therapy, adjuvant TMZ	2 years	EMC

Abbreviations: F = female, IDH = isocitrate dehydrogenase, M = male, MGMT = methylguanine methyltransferase, TMZ = temozolomide, WHO = World Health Organization.

### 2.2 Optimization of MR Acquisition

All patients underwent scanning at both 3 and 7 T MRI. The time between scans was between 1 and 11 days, with an average of 4 days. 3 T scanning was performed at either LUMC or EMC. LUMC scans were performed on a 3 T Philips Ingenia scanner (Philips Healthcare, Best, the Netherlands), where the clinical protocol consisted of a precontrast and postcontrast T_1w_ scan, DWI b0‐1000, ASL perfusion and T_2w_ FLAIR scans. Additionally, QSM, precontrast and postcontrast multiecho SAGE, and SAGE‐DSC images were acquired. EMC scans were performed on a 3 T GE Signa Premier scanner (GE, Milwaukee, WI, USA), where the clinical protocol consisted of precontrast and postcontrast T_1w_, DWI b0‐1000, T_2w_ FLAIR and T_2w_ PROPELLER scans. Additionally, precontrast and postcontrast multi‐echo SAGE^1^ and SAGE‐DSC images were acquired. Lastly, the 7 T images were acquired at LUMC on a 7 T Philips Achieva scanner. The 7 T protocol included a precontrast and postcontrast T_1w_ scan, DWI and SAGE‐DSC scans. The SAGE‐DSC scan parameters varied across patients as part of the protocol optimization. The sequence parameters of SAGE‐DSC for each institute and field strength are shown in Table 2. To mitigate the increased field inhomogeneities at 7 T, an image‐based optimized brain shimming procedure was used to set the third order shim gradients. Echo spacing was minimized within the limits of the gradient performance, given the spatial resolution and SENSE acceleration factor.

**TABLE 2:** Sequence parameters of the SAGE‐DSC sequence at the different hospitals and field strengths. For the 7 T sequence, parameters in bold indicate the optimal values of the final protocol. Note that the second GRE in the Philips sequences are asymmetric, recorded at the same TE (GRE1) after the 180° pulse of the SE.

SAGE‐DSC parameters	3 T EMC	3 T LUMC	7 T LUMC
MR‐system	GE Signa Premier	Philips Ingenia	Philips Achieva
TE (ms) (GRE1/GRE2/SE)	17.2/—/70.0	12.0/57.3/86.1	10.7/51.2/81.6
TR (ms)	1500	1500	1600
FA (°)	90	90	90
Resolution (mm^3^)	2.3 × 2.3 × 3.0	2.5 × 2.5 × 5.0	1.5 × 1.7 × 5.0
Image matrix (x × y)	128 × 128	96 × 96	144 × 144
Number of slices	15	22	11/12
Number of time points	120	60	60/90
Multiband factor	N/A	2	N/A
SENSE factor	2	2.5	2.5
Acquisition time (min)	3:00	1:35	1:41/2:29/3:15
GBCA	Double dose of 0.1 mL/kg Gadovist (5 min apart), with 5 ml/s injection speed and 20 s injection delay.	Single dose of 0.2 mL/kg Clariscan, with 5 mL/s injection speed and 14 s injection delay.	Single dose of 10/15 mL Clariscan, with manual injection estimated at a speed of 2/3 mL/s.

### 2.3 Image Assessment

Normal‐appearing gray matter (NAGM) and white matter (NAWM) masks were obtained in MATLAB R2023b (MathWorks, Natick, MA, USA) using SPM12 (r7771) from the precontrast T_1w_ scans. Specifically, the generated tissue probability maps were normalized and voxels with a probability below 0.8 were discarded from the mask. Subsequently, these masks were rigidly registered to the mean SAGE‐DSC image, after which the voxels contralateral to the tumor served as the normal‐appearing tissue masks (masks shown in Figure S1). Because no motion was observed in any dataset, no motion correction was performed. Then, relative CBV (rCBV) maps were calculated from the gradient‐echo data using in‐house code developed by Arzanforoosh et al. [11], which includes the Boxerman‐Schmainda‐Weisskoff (BSW) leakage correction method [18] and normalization to the average CBV in contralateral NAWM.

The 3 and 7 T rCBV maps were visually compared by assessing the perfusion‐related rCBV intensity in contrast‐enhancing areas relative to contralateral normal‐appearing tissue. Furthermore, the maps were graded by an experienced neuroradiologist (M.S., 16 years of experience) regarding map quality and perfusion intensity on a 4‐point Likert scale (see Table 3). The neuroradiologist was blinded to the field strength and there was a two‐week washout period between the 3 and 7 T maps. This assessment was only done in patients 5–8, as these data were acquired with equal acquisition parameters.

**TABLE 3:** Scoring options for map quality and perfusion intensity. The map was considered not assessable if data were incomplete, incorrectly postprocessed or contained extensive motion artifacts. Lesions were considered measurable if they were contrast‐enhancing lesions with an approximate size of at least 70 mm^2^.

	Map quality		Perfusion intensity
0	Not assessable	0	Neither increased nor decreased
1	Poorly assessable	1	Increased
2	Moderately assessable	2	Decreased
3	Fairly assessable	3	No measurable lesion
4	Good assessable	4	Not assessable

Furthermore, the temporal signal‐to‐noise ratio (tSNR) was computed for the SE and GRE signals as follows:

(1) $$\text{tSNR}=\frac{{\bar{S}}_{\text{baseline}}}{\sigma_{S,\text{baseline}}},$$

with ${\bar{S}}_{\text{baseline}}$ the mean baseline signal magnitude after steady‐state has been reached and $\sigma_{S,\text{baseline}}$the standard deviation of the baseline signal. The contrast‐to‐noise ratio (CNR) was computed as follows [19]:

(2) $$\mathrm{CNR}=\frac{∆R_{2,\max}^{\left(*\right)}}{\sigma_{R_{2}^{\left(*\right)},\text{baseline}}}.$$

The relaxation rates $∆R_{2}$ and $∆R_{2}^{*}$, hereafter referred to as $∆R_{2}^{\left(*\right)}$, were calculated using SE or GRE, respectively, according to:

(3) $$∆R_{2}^{\left(*\right)}\left(t\right)=-\frac{1}{\mathrm{TE}}\ln\left(\;\frac{S\left(t\right)}{{\bar{S}}_{\text{baseline}}}\;\right),$$

with $S\left(t\right)$ the signal intensity at time t. Then, $∆R_{2,\max}^{\left(*\right)}$ was defined as the maximum change in relaxation rate, i.e. $∆R_{2,\max}^{\left(*\right)}=\max_{t}∆R_{2}^{\left(*\right)}\left(t\right)$, and $\sigma_{R_{2}^{\left(*\right)},\text{baseline}}$ as the standard deviation of the relaxation rate at baseline. These image quality metrics were computed per voxel and then averaged over the NAWM or NAGM.

### 2.4 Signal behavior simulations

To investigate the feasibility of VSI and VAI at 7T, the behavior of $\Delta R_{2}^{\left(*\right)}$ was first simulated and then analyzed in vivo. An open‐source 2D simulation model [20] was used to evaluate the sensitivity and specificity of GRE and SE for varying vessel sizes. The model incorporates inflow, leakage and diffusion of contrast agent, water diffusion, T_1_ and T_2_ relaxation and susceptibility effects (see Van Dorth et al. [21] for a more extensive description). GBCA relaxivities r_1_/r_2_ were defined as 3.3/4.8 s^‐1^ mM^‐1^ for 3T and 3.0/4.3 s^‐1^ mM^‐1^ for 7T [22]. Simulations were performed using the sequence settings from the Philips MR‐systems, i.e. TE (GRE/SE) = 12/86 ms and TR = 1500 ms for 3T and TE (GRE/SE) = 11/82 ms and TR = 1600 ms for 7T. Similar experiments as described in Boxerman et al. [8] were performed. The passage of a single dose of GBCA was simulated using Parker’s arterial input function [23]. Furthermore, a CBV of 2% was adopted (typical for white matter [24]), with vessel radii varying between 2‐150 μm. Finally, the behavior of $∆R_{2,\max}^{\left(*\right)}$ as a function of vessel size was compared between 3T and 7T in terms of vessel size sensitivity and specificity.

### 2.5 Vessel Size Imaging

First, the apparent diffusion coefficient (ADC) maps as obtained from DWI were rigidly registered to the mean SAGE‐DSC image (DWI image matrix at 3T EMC: 256 x 256 x 49, resolution: 0.9 x 0.9 x 3 mm^3^; at 3T LUMC: image matrix: 256 x 256 x 27, resolution: 0.9 x 0.9 x 5 mm^3^; at 7T LUMC: image matrix: 192 x 192 x 24, resolution: 1.2 x 1.2 x 5 mm^3^). Then, VSI maps were obtained following Kiselev et al. [9] and using the aforementioned in‐house code, in which the mean vessel size was calculated according to:

(4) $$\text{Vessel}\;\text{Size}=0.867\;{\left(\mathrm{CBV}\cdot \mathrm{ADC}\right)}^{\frac{1}{2}}\;\left[\frac{∆R_{2}^{*}}{{\left(∆R_{2}\right)}^{\frac{3}{2}}}\right].$$

Voxels with a signal drop fewer than 2 standard deviations from the mean baseline signal in the NAWM were disregarded to ensure only areas with GBCA‐induced signal changes were included. The 3T and 7T VSI maps were visually compared to each other. The mean estimated vessel size and the vessel size ratio of NAGM to NAWM were compared in a case‐by‐case fashion.

### 2.6 Vascular Architectural Imaging

Hysteresis loops were obtained by plotting $∆R_{2}^{*}$ and $∆R_{2}^{3/2}$ against each other for both field strengths [4, 12]. First, hysteresis loops were generated using the signal behavior simulations. Because factors such as bolus arrival time and oxygenation were kept constant, the 3 and 7 T simulated loops were compared in terms of slope and length of the long axis, disregarding changes in loop area and direction. Second, hysteresis loops were generated using the SAGE‐DSC data from NAGM and NAWM separately. These loops were compared between 3 and 7 T in terms of slope, length of the long axis, loop area and direction.

## 3 Results

### 3.1 Protocol Optimization

**FIGURE 1:**
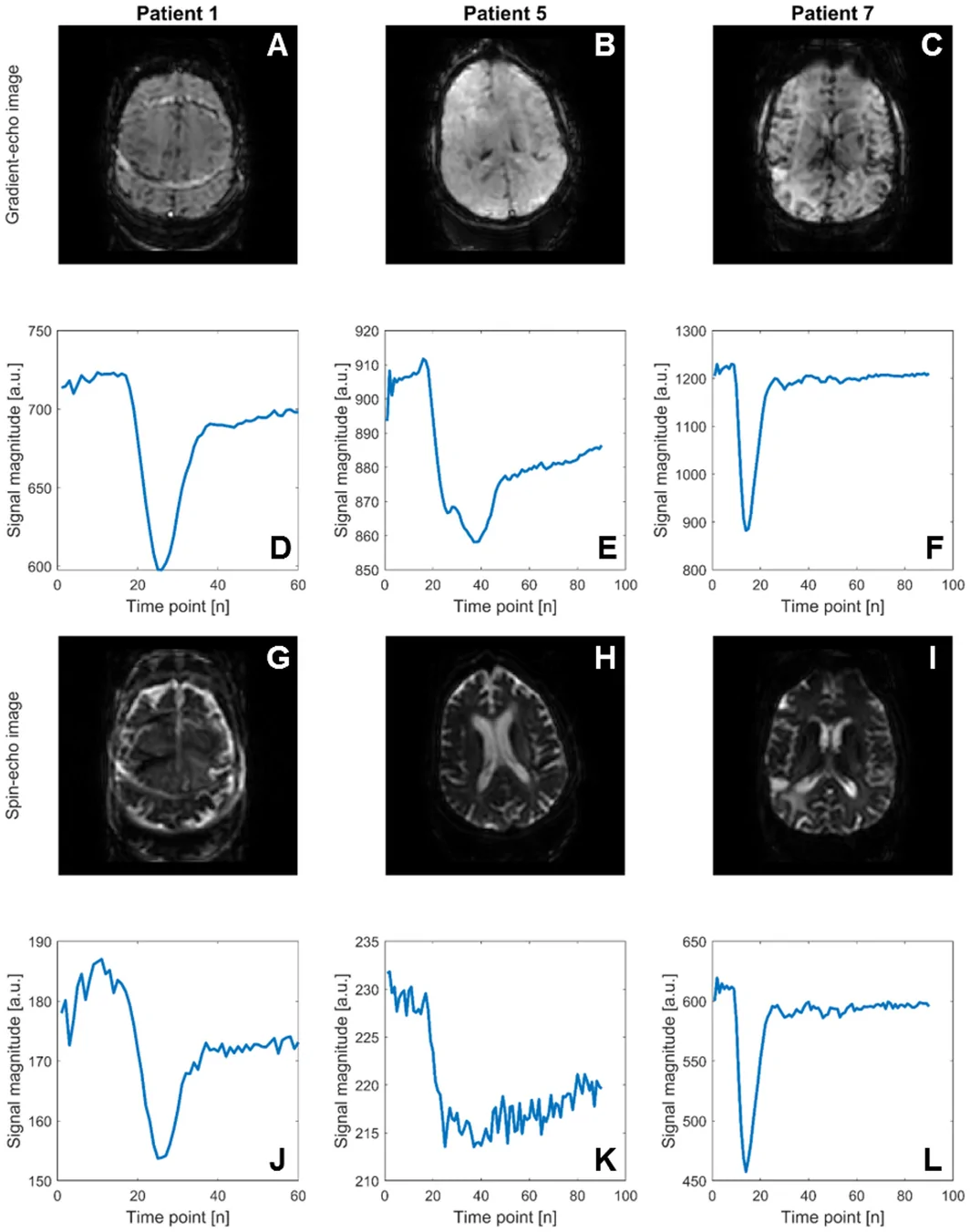
Example SAGE‐DSC data for patient 1 (left), patient 5 (middle) and patient 7 (right), illustrating the effect of protocol changes. The first and third rows show a slice from the gradient‐ and spin‐echo image data, while the second and fourth rows show the averaged signal time courses over the NAGM, respectively. Note the broad and relatively shallow bolus passage in patient 1 (D, J), and the effect of the IV placement in the wrist instead of elbow on the bolus passage in patient 5 (E, K). The image quality and shape of the signal time courses were best for patient 7 (F, L), where a lower dose was injected with a higher injection speed compared to the other two patients. An overview of all patients can be found in Figure S2.

Table 4 provides an overview of the protocol parameters of each 7 T SAGE‐DSC scan. Figure 1 shows examples of the image data and the average signal time courses in NAGM for patients 1, 5 and 7, while an overview of all patients can be found in Figure S2. In patients 1 and 2, 15 mL GBCA was used following the standard dosages at 3 T. However, the averaged signal time courses in these patients showed a broad peak and no full signal recovery to baseline. A varying signal drop of 6%–20% was observed for GRE and 6%–44% for SE in the NAGM. The bolus duration, measured from the start of the signal drop until the steady postbolus level, was 20–30 s.

**TABLE 4:** Overview of scan parameters and GBCA injection scheme per patient at 7 T.

#	Echo times	# of slices	# of time‐points	Scan time	GBCA dosage	Estimated injection speed
1	8.4/46.2/76.2 ms (TE1/TE2/TE3 = SE)	12	60	1:41 min.	15 mL	2 mL/s
2	12.2/53.8/83.9 ms (TE1/TE2/TE3 = SE)	11	60	1:41 min.	15 mL	2 mL/s
3	10.5/40.9/81.2/111.6/142.0 ms (TE1/TE2/TE3/TE4/TE5 = SE)	11	60	3:15 min.	10 mL	2 mL/s
4	12.2/53.9/83.9 ms (TE1/TE2/TE3 = SE)	11	90	2:29 min.	10 mL	3 mL/s
5	10.7/51.2/81.6 ms (TE1/TE2/TE3 = SE)	11	90	2:29 min.	10 mL	2 mL/s (IV in wrist)
6	10.7/51.2/81.6 ms (TE1/TE2/TE3 = SE)	11	90	2:29 min.	10 mL	3 mL/s
7	10.7/51.2/81.6 ms (TE1/TE2/TE3 = SE)	11	90	2:29 min.	10 mL	3 mL/s
8	10.7/51.2/81.6 ms (TE1/TE2/TE3 = SE)	11	90	2:29 min.	10 mL	3 mL/s

Reducing the GBCA dose to 10 mL and increasing the injection speed to an estimated 3 mL/s, the signal time curves showed a sharper bolus passage and more signal recovery towards baseline (Figure 1F,L). With this improved injection protocol, the signal drop was 21%–36% for GRE and 17%–28% for SE. Furthermore, bolus transit time decreased to approximately 16 s. Improved image quality was observed in patient 7 compared to patient 1. However, in patient 5 (who received this reduced dose), atypical signal behavior was observed (Figure 1E,K), which might be related to the intravenous (IV) access placed at the wrist instead of the elbow. Furthermore, the data of patient 3 was inadvertently obtained with additional echoes, leading to a long TE and thus low signal intensity for the SE. Lastly, patient 6 exhibits signal loss in the center of the brain (Figure S2).

### 3.2 Image Assessment

**FIGURE 2:**
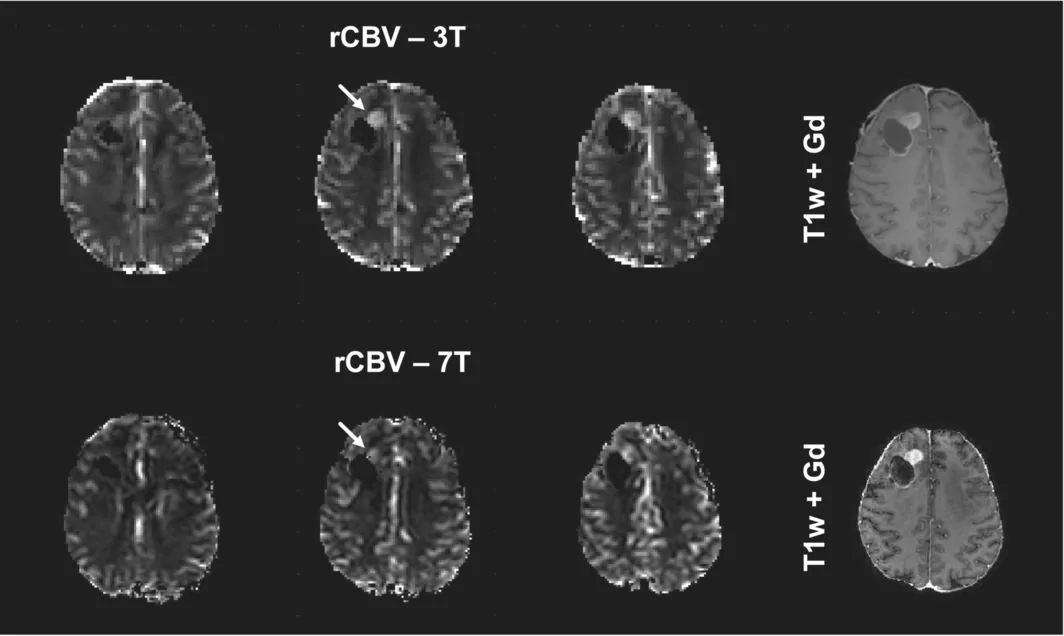
Three slices of the rCBV maps derived from the gradient‐echo of the SAGE‐DSC data at 3 T (top) and 7 T (bottom) for patient 2. On the right, a slice from the T1w postcontrast scan is shown at 3 T (top) and 7 T (bottom), approximately corresponding to the middle slice of the perfusion maps. The patient showed contrast enhancement around the resection area with increased perfusion (white arrows).

To investigate whether 3 and 7 T SAGE‐DSC yields similar perfusion information, rCBV maps from both field strengths were compared. Figure 2 shows an example of a patient where contrast enhancement could be observed on the postcontrast T_1w_ scan around the resection area, accompanied by elevated perfusion on both 3 and 7 T rCBV maps.

**FIGURE 3:**
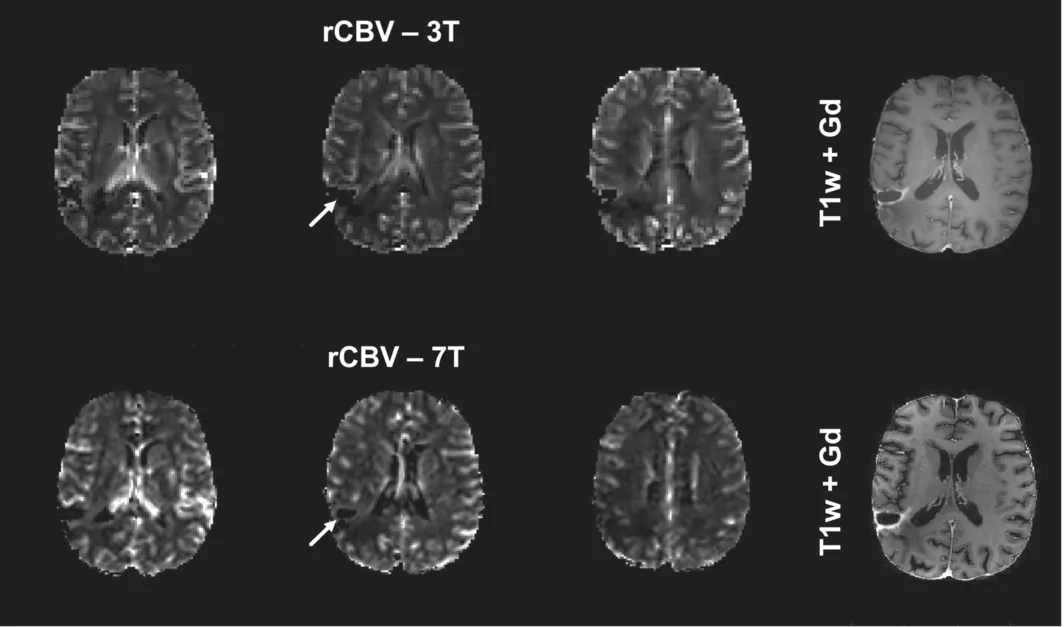
Three slices of the rCBV maps derived from the gradient‐echo of the SAGE‐DSC data at 3 T (top) and 7 T (bottom) for patient 7. On the right, a slice of the T1w postcontrast scan is shown at 3 T (top) and 7 T (bottom), approximately corresponding to the middle slice of the shown perfusion maps. The patient showed a minor increase in enhancement and no elevated perfusion (white arrows).

Figure 3 shows an example where the postcontrast T_1w_ scan showed a slight increase in enhancement near the resection area, while the rCBV maps showed a decrease in perfusion at both field strengths.

The results of the quality and perfusion assessment are shown in Table 5. Between 3 and 7 T, the map quality assessment largely agreed (3/4 cases) while more discrepancies were present in the perfusion intensity assessment (2/4 cases). For patient 5, the lesions were outside of the field of view at 7 T and therefore not assessable. For patient 6, the lesion was very difficult to localize on the postcontrast T1w‐scan, leading to high uncertainty in this assessment at 7 T. An additional similarity assessment between the 3 and 7 T rCBV maps (1 = *no similarity*, 2 = *low similarity*, 3 = *moderate similarity*, 4 = *high similarity*) showed that there was moderate similarity for patient 5 and high similarity for patients 6–8.

**TABLE 5:** Assessment of the 3 and 7 T rCBV maps per patient in terms of map quality and perfusion intensity.

Patient	Map quality		Perfusion intensity	
	3 T	7 T	3 T	7 T
5	Good	Fair	Decreased	Not assessable
6	Good	Good	Decreased	Increased
7	Good	Good	Increased	Increased
8	Good	Good	Decreased	Decreased

**FIGURE 4:**
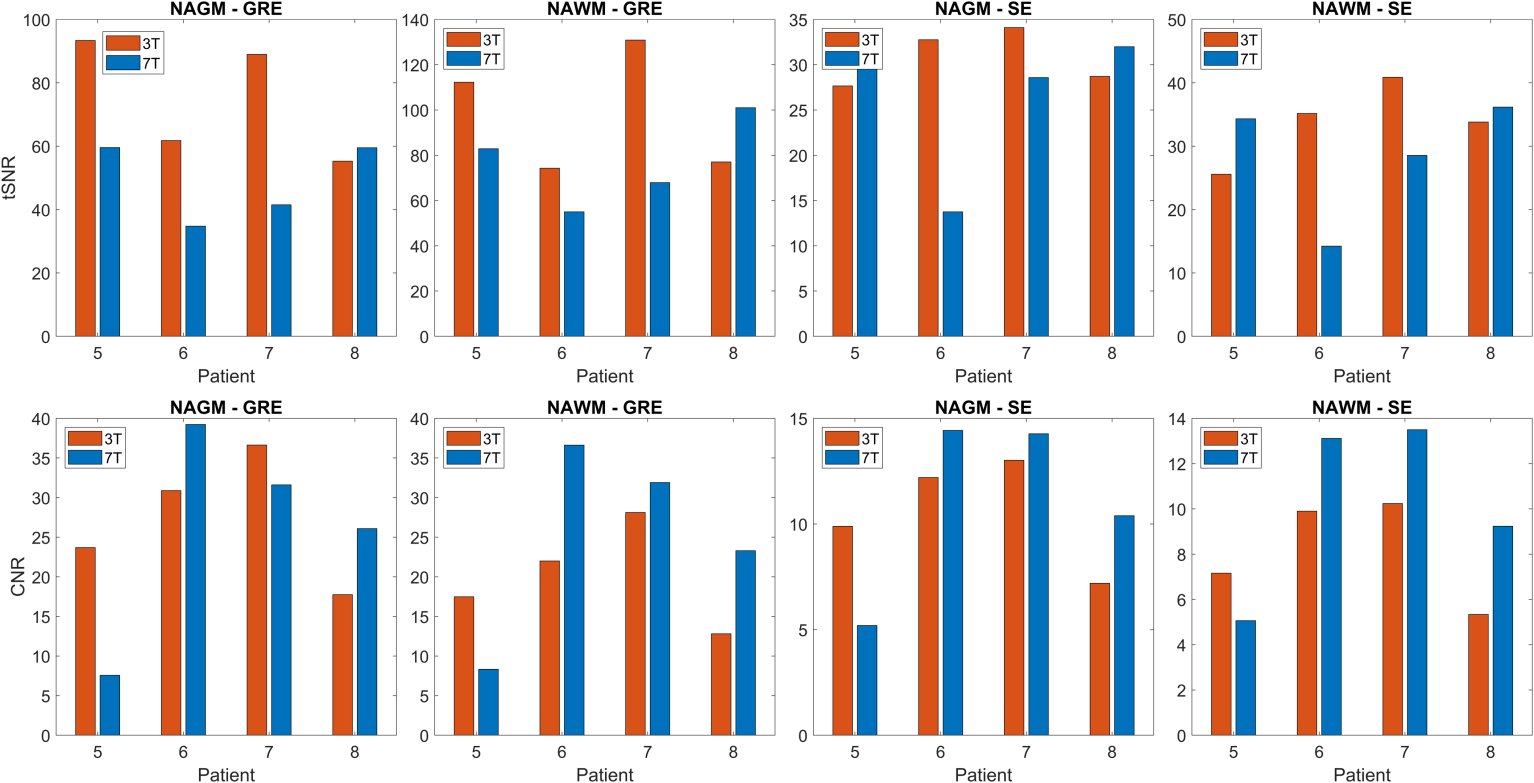
tSNR (top) and CNR (bottom) measurements at 3 T (red) and 7 T (blue). Results are stratified by echo (GRE on the left, SE on the right) and by tissue (NAGM in first and third columns, NAWM in second and fourth columns). While the tSNR was generally higher at 3 T, the CNR was often higher at 7 T.

The tSNR and CNR measurements are shown in Figure 4 for patients 5–8, while a complete overview can be found in the appendix (Figure S3). For most patients, the tSNR was higher at 3 T than 7 T, whereas the reverse held for the CNR. For patient 5, the effect of the IV‐placement is reflected in the low CNR at 7 T.

### 3.3 Signal Behavior Simulations

**FIGURE 5:**
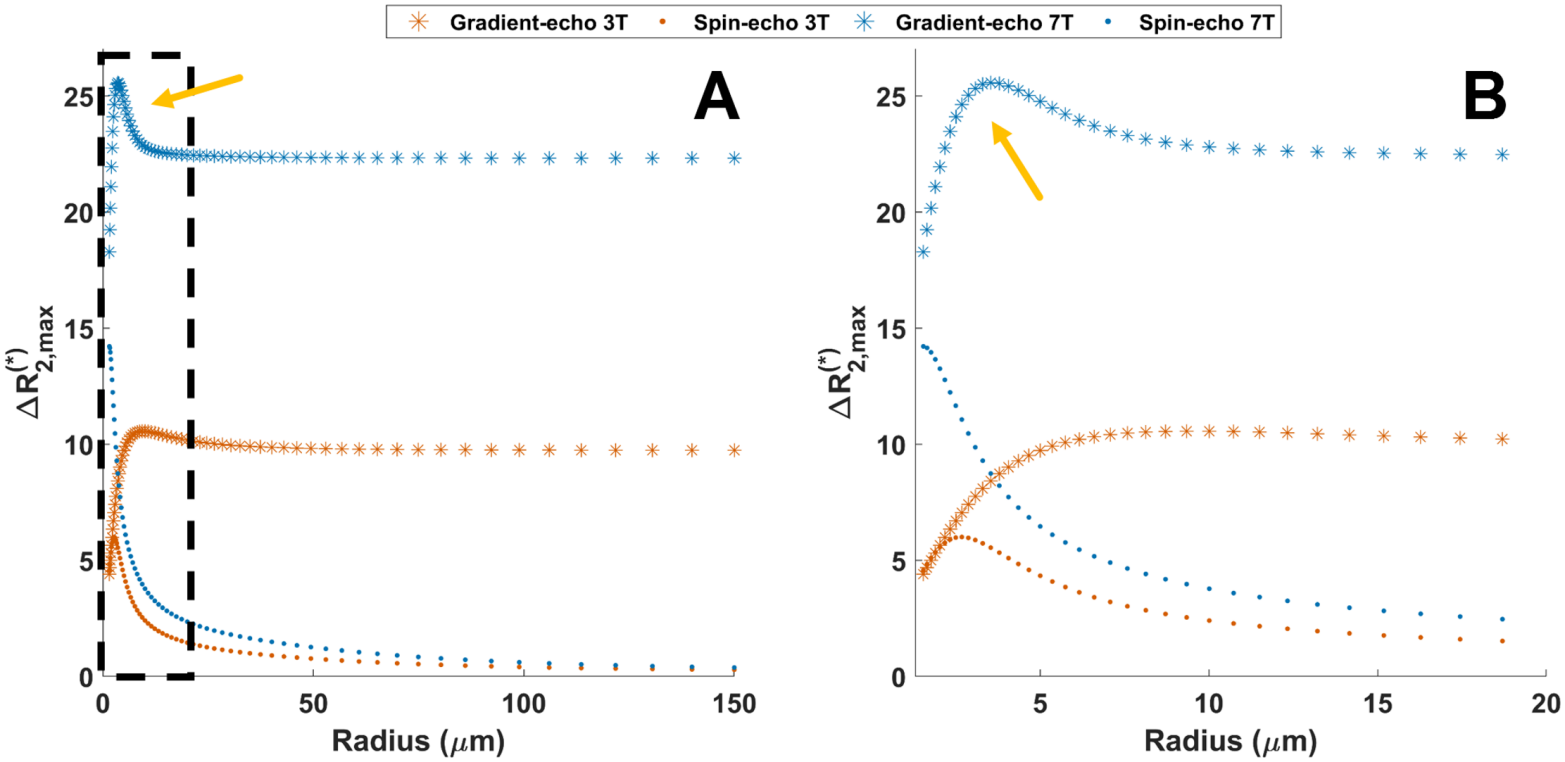
Change in relaxation rate over a range of vessel sizes simulated at 3 and 7 T for the SAGE‐DSC sequences from the Philips systems. B (right) shows a zoomed‐in version of the figure as delineated by the dashed box in A (left). At 7 T, GRE starts to exhibit specificity towards the microvasculature as well (yellow arrows).

Figure 5 shows the behavior of GRE and SE signal changes as function of vessel size, simulated for 3 and 7 T. It confirms the expected higher sensitivity of GRE (i.e., a large $∆R_{2,\max}^{*}$ across a broad range of vessel sizes), and a clear specificity of SE (large $∆R_{2,\max}$ only for small vessel sizes). The SE specificity appeared to shift towards smaller radii for increasing field strength. Interestingly, both SE and GRE seemed to become more specific to smaller vessels with increasing field strength. Indeed, the plateau for GRE only established after a small peak at 7 T (yellow arrow). Note that the 7 T curves were obtained according to the final protocol, that is, at two‐thirds of the GBCA dose at 3 T.

**FIGURE 6:**
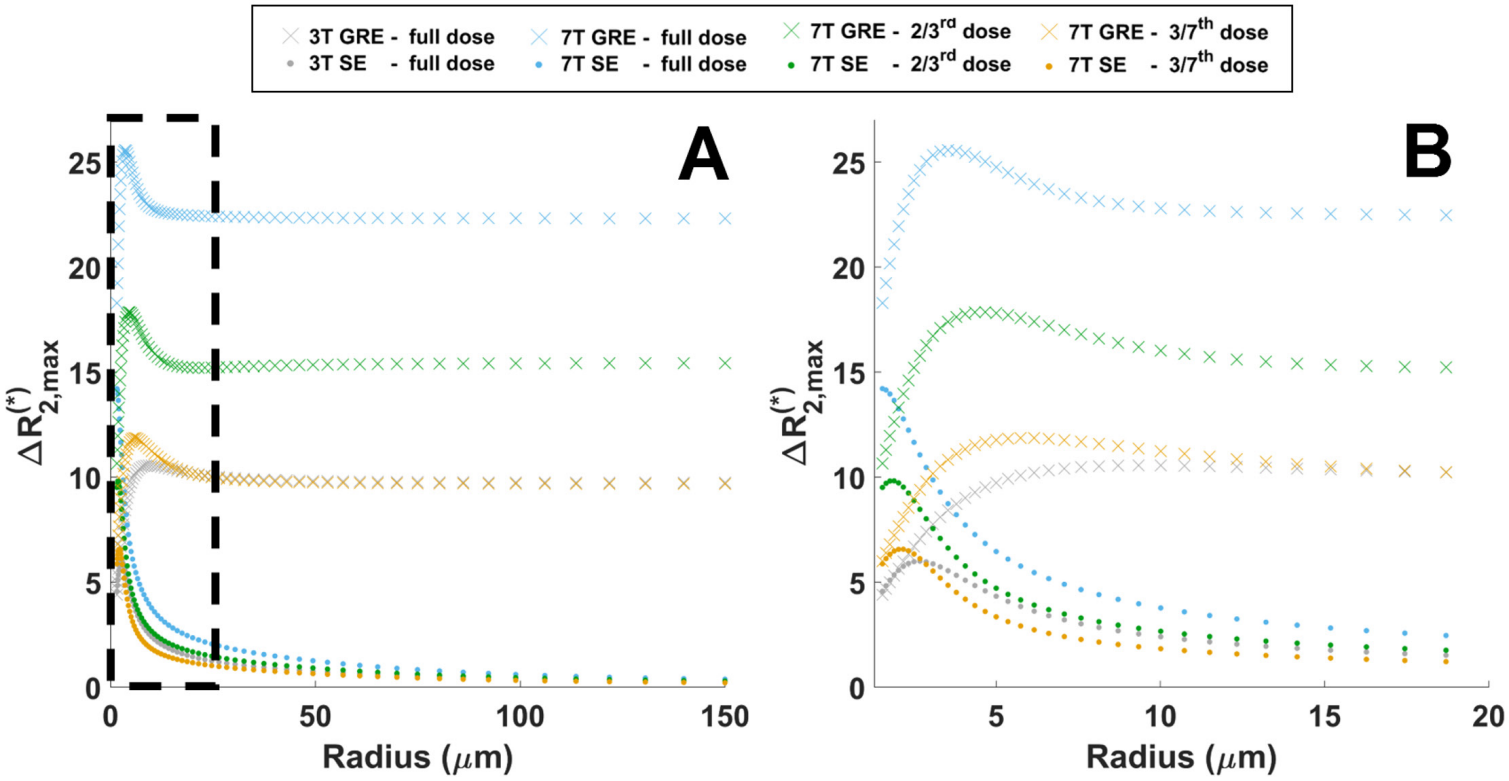
Change in relaxation rate over a range of vessel sizes simulated at 7 T for different amounts of contrast agent (compared to the standard dosage at 3 T, shown in gray for reference). Again, B (right) shows a zoomed‐in version of the figure as delineated by the dashed box in A (left). As the contrast agent dosage decreases, the sensitivity and specificity of the GRE and SE signals, respectively, decrease as well.

Figure 6 shows the simulated behavior of the GRE and SE signal changes at 7 T for different dosages of contrast agent. Notably, the 7 T curves at 3/7ths of the standard dose show similar behavior as the 3 T curves (in gray) at the full standard dose in the static dephasing regime.

### 3.4 Vessel Size Imaging

**FIGURE 7:**
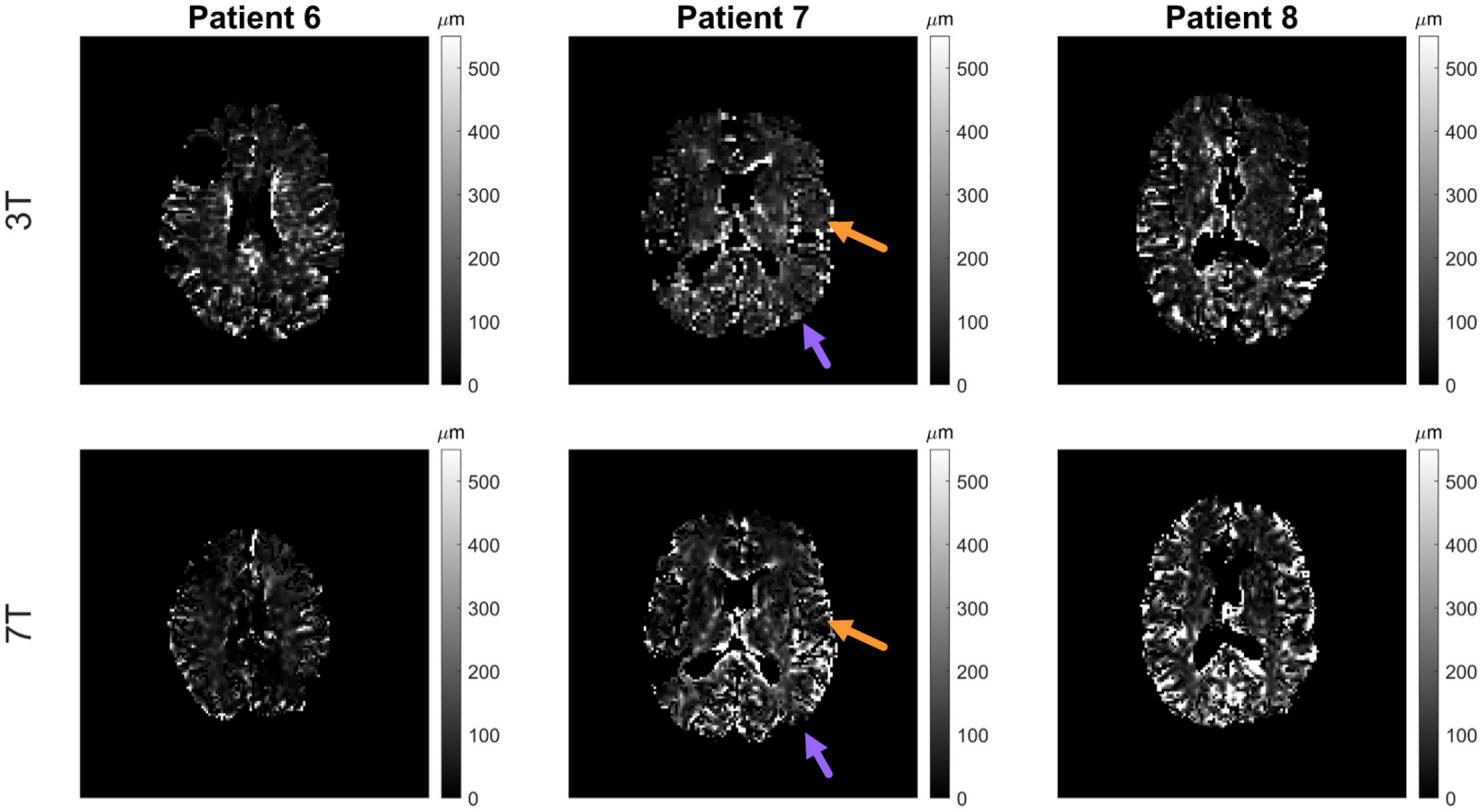
VSI maps for patients 6 (left), 7 (middle) and 8 (right), both at 3 T (top) and 7 T (bottom). A similar pattern can be observed at 3 and 7 T. The orange arrows point at an example in which details are better discernible on the 7 T map due to the increased resolution. The purple arrows point at an example where the vessel size index could not be computed on the 7 T map while this was possible on the 3 T map.

Figure 7 shows a representative slice at approximately equal locations for patients 6–8 at 3 and 7 T. An overview of all patients is given in Figure S4 for 3 T and S5 for 7 T, which includes rCBV, ADC and Q maps. In general, the vessel size pattern appeared similar at 3 and 7 T. The 7 T maps had a higher resolution compared to 3 T, making smaller structures better discernible (orange arrows). Nevertheless, more voxels had too small a signal drop to be included in the VSI calculations at 7 T (purple arrows).

Averaging over all patients except 1, 3 and 5 (due to artifacts, incorrect number of echoes and deviating IV‐placement, respectively), the NAGM‐to‐NAWM ratio of rCBV values at 3 T was 1.6 and 1.4 based on GRE and SE data, respectively. At 7 T, these ratios were 1.3 and 1.2, respectively. The NAGM‐to‐NAWM ratio of VSI values was 1.4 at both 3 and 7 T. The estimated vessel size at 3 T was 104 ± 106 μm in NAGM and 77 ± 70 μm in NAWM. At 7 T, the estimated vessel size was 55 ± 69 μm in NAGM and 49 ± 58 μm in NAWM. The distribution of vessel sizes is skewed (see Figures S6 and S7). The mode, calculated as the maximum in the probability density function of the kernel density estimate, was 55 μm in NAGM and 56 μm in NAWM at 3 T and 45 μm in NAGM and 44 μm in NAWM at 7 T.

### 3.5 Vascular Architectural Imaging

**FIGURE 8:**
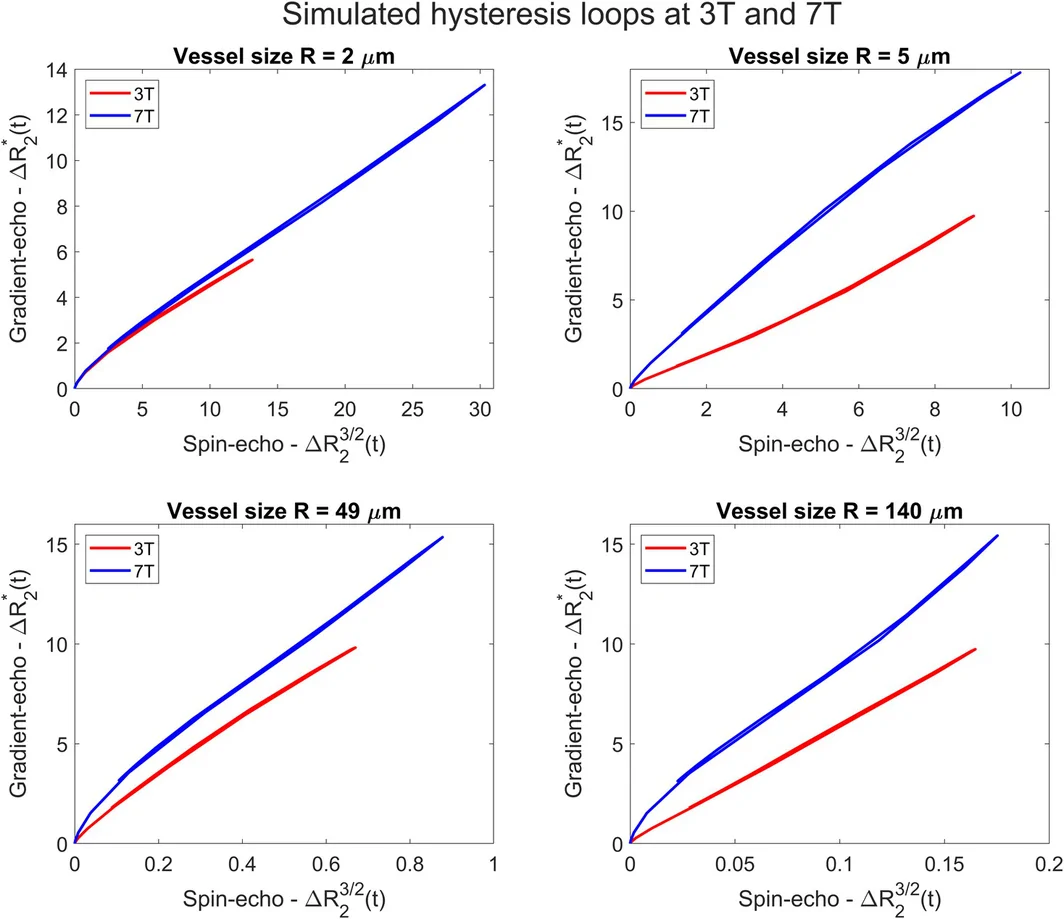
Hysteresis loops generated using the signal behavior simulations at 3 T (red) and 7 T (blue) for different vessel sizes. The GBCA dose at 7 T was simulated according to the final protocol, and thus at 2/3 of the dose at 3 T.

The simulated hysteresis loops can be found in Figure 8 for varying vessel sizes. In all cases, the loops extended further and have a steeper slope for 7 T than 3 T. As expected, peak $∆R_{2}$ decreases quickly, while peak $∆R_{2}^{*}$ stays roughly the same as the vessel size increases.

**FIGURE 9:**
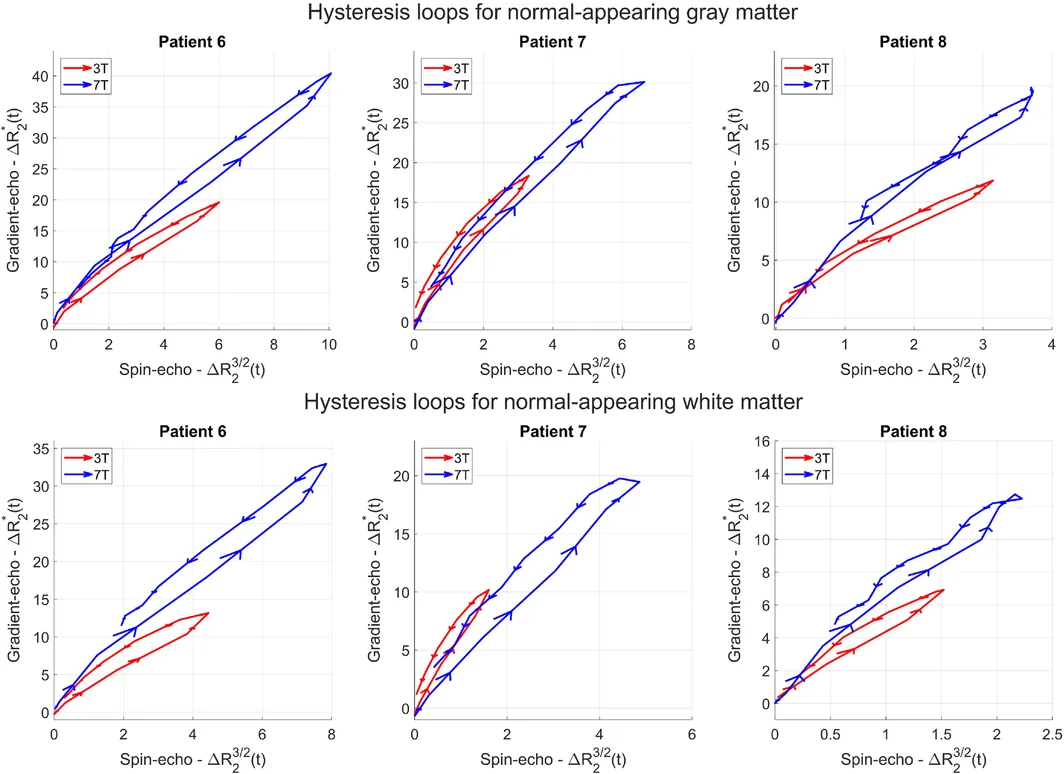
Hysteresis loops derived from averaged GRE and SE signal in normal‐appearing gray matter (top) and white matter (bottom) for patient 6 (left), patient 7 (middle) and patient 8 (right). The loops are shown for 3 T (red) and 7 T (blue) and span the duration of the bolus passage.

Next, hysteresis loops were generated with the SAGE‐DSC data. Again, Figure 9 shows the loops for patients 6–8 in NAGM (top) and NAWM (bottom), while an overview of all patients can be found in Figure S8. The loops showed a comparable shape and counter‐clockwise direction at both 3 and 7 T, with a larger peak $∆R_{2}^{\left(*\right)}$ at 7 T. For patient 7, a steeper slope was observed in the 3 T loops than the 7 T loops. Conversely, the loops of patients 6 and 8 displayed steeper slopes at 7 T, in line with the simulated loop behavior.

## 4 Discussion

The first aim of this work was to establish a protocol for SAGE‐DSC at 7 T. In terms of imaging parameters, we found that the following settings provided a good trade‐off between temporal resolution and coverage: TE (GRE1/GRE2/SE) = 10.7/51.2/81.6 ms and TR = 1600 ms, with 90 time points acquired at a resolution of 1.5 × 1.7 × 5.0 mm^3^ for 11 slices. Compared to 3 T, the in‐plane resolution improved by 52%–56% in the 7 T sequence. Furthermore, administration of 10 mL 0.5 M gadoterate meglumine (two‐thirds of the standard 3 T dose) with an estimated injection speed of 3 mL/s provided good data quality. Images obtained with the higher‐dose protocol suffered from image artifacts. Note that the dosage was not adjusted to the patient's body weight, which can lead to differences in GBCA concentration in the blood. Indeed, this might explain the signal loss observed in patient 6 (see Figure S2); due to the patient's relatively lower body mass, increased GBCA concentrations might have caused the increased susceptibility artifacts.

The second aim of this work was to evaluate the feasibility of microvascular characterization at 7 T using SAGE‐DSC. Image assessment by an experienced neuroradiologist showed that the rCBV maps were of similar quality between 3 and 7 T, while the perfusion intensity assessment showed more discrepancies (2/4 cases). In the first case, the lesions were outside of the field of view and thus not assessable at 7 T. In the second case, the lesion was difficult to localize on the T1w postcontrast scan; the SAGE‐DSC scan was highly angulated, and it was not possible to make overlays of the rCBV map and the postcontrast T1w scan. This probably led to a mismatch in lesion location between 3 and 7 T and hence a discrepancy in the perfusion assessment. Still, the rCBV maps were found to have a high similarity between 3 and 7 T.

At 7 T, CNR improved (Figure 4). Counterintuitively, tSNR was higher at 3 T than 7 T for most patients. However, tSNR is affected by a multitude of factors, including voxel size (which decreases up to more than half when going from 3 to 7 T) [25] as well as a higher contribution of physiological noise at 7 T [26]. Nevertheless, for rCBV and VSI estimations, CNR is a more relevant metric than tSNR [19].

Next, simulations were performed to investigate the behavior of SE and GRE at both field strengths for different vessel sizes. In accordance with Boxerman et al. [7], we found a broad sensitivity for GRE and a higher microvascular specificity for SE. As expected, $∆R_{2}^{\left(*\right)}$ was higher for 7 T than 3 T due to increased local field perturbations. Interestingly, SE specificity shifted towards smaller vessel sizes with increasing field strength. While Troprès et al. [8] reported an SE peak at 5 μm for 1.5 T, we found a peak at 3 μm for 3 T and 1.5 μm for 7 T. We hypothesize that this is caused by a shift in the cross‐over of the motional narrowing regime (MNR) and static dephasing regime (SDR) [9, 27]: This is where the specificity of the SE occurs. Motional narrowing occurs when $\delta \omega t_{D}\ll 1$, with tD=r2D the time of diffusion across the vessel and $\delta \omega =2\pi \chi \gamma B_{0}$ the Larmor frequency shift. In words, motional narrowing occurs when the vessel size is small enough such that a spin can diffuse past many vessels. Then, each spin experiences the full range of field distortions and thus acquires a similar net phase. These spin history effects cannot be refocused, which results in a similar relaxation change for both GRE and SE. On the other hand, static dephasing occurs when $\delta \omega t_{D}\gg 1$, that is, when the diffusion distance is small compared to the vessel size. Then, each spin experiences different local field perturbations, leading to static dephasing. This dephasing can be refocused by the 180° pulse of SE, but not by GRE. Thus, $∆R_{2}$ decreases for larger vessel sizes, while $∆R_{2}^{*}$ does not. When moving from 3 to 7 T, δω increases and the cross‐over of the regimes will occur at a smaller $t_{D}$. With D a physiological constant, a smaller $t_{D}$ occurs at a smaller r, and thus the specificity arises at smaller vessel sizes.

This observed shift in specificity is an important finding for the potential clinical application of SAGE‐DSC at 7 T. Indeed, SE‐DSC signals will mainly reflect vessels with a radius around 1.5–2.0 μm at 7 T. Because the smallest vessels in the brain have a radius between 1.0 and 2.5 μm, 7 T VSI maps could provide more information on those vessels that are less well‐captured in 3 T VSI maps. However, it should be noted that the GBCA dose was lowered in the final protocol. The simulations in Figure 6 show that a reduction to 3/7ths of the standard dosage leads to vessel size sensitivities at 7 T similar to those at 3 T. This might imply that dose reduction can be achieved without losing vascular contrast with increasing field strength. Indeed, we found that the CNR remained higher at 7 T despite reduced GBCA doses (Figure 4). Interestingly, GRE also started to exhibit specificity towards smaller vessel sizes at 7 T. Similar behavior has been observed before [28, 29]. We hypothesize that this can also be explained by motional narrowing: with stronger local field perturbations, motional narrowing might be less complete, leading to more variations in phase offsets and thus larger $∆R_{2}^{*}$.

The estimated rCBV was higher in NAGM than NAWM at both field strengths, which is consistent with literature [13, 30]. At 7 T, NAWM rCBV was 1.3 times lower than NAGM rCBV for GRE and 1.2 times for SE. Elschot et al. [17] obtained similar results with SE‐DSC at 7 T, where the ratio of cortical‐GM‐to‐WM CBV was found to be 1.28 ± 0.11. The estimated VSI was higher in NAGM than NAWM at both field strengths. While the mean estimated vessel sizes were larger than described in literature [9, 31, 32], the histograms (Figures S6 and S7) showed that this was in large part caused by the skewed distribution of vessel sizes. Furthermore, it is important to note the semiquantitative nature of VSI, the dependence on the choice of ROI and the small sample size of the study. The mean estimated vessel size was larger at 3 T than 7 T. This agrees with the simulations from Figure 5, in which the sensitivity and specificity of GRE and SE shifts towards smaller vessel sizes at 7 T.

The general shape of the hysteresis loops was similar for both field strengths, yet they extended further for 7 T than 3 T. This is in agreement with the simulations and can be explained by the increased susceptibility effects at higher field strengths, which were only partially mitigated by the reduced GBCA dose. Furthermore, the loops for NAGM extended further than those of NAWM at both field strengths, reflecting the higher capillary density of gray matter [33]. The counter‐clockwise direction of the curves points towards capillary or venule dominance [12]. As limited tumor tissue was present in these postoperative patients and SAGE‐DSC data was acquired at a single time point, we restricted our analysis to NAGM and NAWM. ROI‐based analyses might provide additional information in preoperative patients or longitudinal studies, for instance to investigate therapy‐induced microvascular alterations and their effect on VAI at 7 T [34].

To the best of our knowledge, this is the first time that SAGE‐DSC has been acquired at 7 T. Knutsson et al. [13] were the first to show the feasibility of DSC‐MRI at 7 T using single‐shot GRE‐EPI. Since then, Buntrock et al. [14] showed that perfusion metrics and high‐resolution perfusion maps can reliably be obtained with GRE‐DSC at 7 T. Their protocol used half of the standard GBCA dose at 3 T. The temporal and spatial resolution was similar to our final protocol, although they achieved an improved resolution in the slice direction. The increased resolution at 7 T is beneficial for its potential clinical application. Furthermore, Elschot et al. [17] investigated SE‐DSC at 7 T, demonstrating the ability to quantify microvascular perfusion in older adults [17]. However, the study did not have a gold standard reference per patient and used literature values instead. While we obtained a higher temporal resolution, our protocol was limited to a 7‐cm coverage. Conversely, Elschot et al. [17] obtained whole‐brain coverage. This trade‐off between spatial and temporal resolution always needs to be reconsidered, depending on the (extent of the) pathophysiology. Lastly, Elschot et al. used a contrast dose of 7 mL 1 M gadobutrol injected at a speed of 5 mL/s with a power injector. In contrast, in our study, only manual injection was possible, resulting in a lower and less‐controlled injection speed of approximately 3 mL/s. Furthermore, the dosage in our study was slightly lower and a different contrast agent was used. Our results support the recommendation for a reduced dose at 7 T. In addition, SAGE‐DSC was acquired without a preload, which is relevant for clinical practice considering the promising findings of other studies [35, 36, 37] that focused on reducing contrast agent dosage.

### 4.1 Limitations

Our study has some limitations. First, the manual injection of GBCA at 7 T limited the injection speed and gave rise to variations between patients. We had to fall back on manual injection as no 7 T‐compatible power injector was available at our site. In addition, no multiband option was available on our 7 T system, while this could be employed at 3 T. Therefore, less coverage could be achieved at 7 T compared to the full‐brain coverage at 3 T. Still, the final protocol for 7 T yielded similar perfusion maps with larger coverage than its 3 T counterpart at EMC.

Second, the separate export of SE and GRE data proved to be challenging, especially on the 3 T system. As a result, the BSW leakage correction package of our clinical software failed, and we resorted to in‐house code for the generation of the rCBV maps. Such practical issues hinder clinical adoption of SAGE‐DSC, as locally manufactured postprocessing software often lacks the certification needed for clinical use.

Lastly, the acquired dataset was very heterogeneous: patients with different clinical characteristics were studied using different injection schemes and scan parameters, with clinical reference data acquired at scanners from different vendors. Partly, this heterogeneity is inherent to the process of protocol optimization. Importantly, though, the results are applicable to a clinical patient population and are not limited to simulations only. Building on this optimized protocol, future research in a larger patient cohort would allow for an analysis stratified by tumor type and treatment regime.

## 5 Conclusions

This feasibility study identified a suitable scan protocol for combined SAGE DSC‐MRI at 7 T, which includes manual injection of 10 mL 0.5 M gadolinium‐based contrast agent at 3 mL/s (a reduction compared to the standard 3 T dose). Furthermore, our study has shown the potential of microvascular characterization using SAGE‐DSC at 7 T. These findings establish a foundation for the implementation of SAGE‐DSC at 7 T and encourage further validation and exploration of diagnostic applications, such as preoperative tumor characterization.

## Author Contributions

**Karen N. van der Werff:** study design, data acquisition, analysis and interpretation of data, drafting of manuscript. **Daniëlle van Dorth:** study design, data acquisition, analysis and interpretation of data, drafting of manuscript. **Krishnapriya Venugopal:** data acquisition, review and editing. **Esther A. H. Warnert:** study design, review and editing. **Dirk H. J. Poot:** study design, interpretation of data, review and editing. **Frans M. Vos:** study design, interpretation of data, review and editing. **Marion Smits:** study design, radiological assessment, review and editing. **Stefan Klein:** study design, review and editing. **C. Chad Quarles:** software for data acquisition, review and editing. **Juan A. Hernandez‐Tamames:** study design, review and editing. **Johan A. F. Koekkoek:** study design, review and editing. **Jeroen de Bresser:** study design, review and editing. **Matthias J. P. van Osch:** study design, interpretation of data, review and editing.

## Funding

The project ‘Vascular Signature Mapping of Brain Tumor Genotypes’ (Project Number 17079) of the open technology research program of Applied and Engineering Sciences is (partly) financed by the Dutch Research Council (NWO) and the Medical Delta program ‘Cancer Diagnostics 3.0.’

## Conflicts of Interest

Marion Smits received consultancy fees (paid to the institution, no direct relation to the presented work) from Bracco. C.J. Gorter MRI center receives research support from Philips. Matthias J.P. van Osch is an unpaid member of the clinical trial steering committee of the cAPPricorn trial of Alnylam. Stefan Klein is scientific director, Dirk H.J. Poot is lab director and Juan A. Hernandez Tamames and Marion Smits are co‐applicants of the ICAI lab ‘Trustworthy AI for MRI,’ a public‐private research program partially funded by General Electric Healthcare (payment to institution). The project ‘Gadolinium‐Free Enhancement MRI’ from Juan A. Hernandez Tamames is partially funded by General Electric Healthcare. C. Chad Quarles receives grant support by the National Institutes of Health through NIH/NCI UH3CA247606.

## Supporting information


**Figure S1:** A representative gradient‐echo image slice and the corresponding NAGM and NAWM masks per patient (rows) and field strength (columns 1–3: 3 T, columns 4–6: 7 T).
**Figure S2:** A representative gradient‐echo image slice and the averaged NAGM signal time course for all patients for the first gradient‐echo (first and second column) and for the spin‐echo (third and fourth column) at 7 T. Note the effect of the reduced GBCA dosage (patients 1–2 vs. patients 3–8) and the effect of the IV access placed in the wrist instead of the elbow (patient 5).
**Figure S3:** tSNR (left) and CNR (right) measurements at 3 T (red) and 7 T (blue). Results are stratified by echo (GRE on the top, SE on the bottom) and by tissue (NAGM in first and third rows, NAWM in second and fourth rows). While the tSNR was generally higher at 3 T, the CNR was often higher at 7 T.
**Figure S4:** Parameter maps for all patients obtained with the 3 T data. From left to right, the first and second columns show the rCBV maps obtained with the spin‐echo and gradient‐echo data, respectively. The third column shows the ADC map. The fourth column shows the so‐called Q map, where Q=∆R2*∆R23/2. Lastly, the fifth column shows the VSI maps.
**Figure S5:** Parameter maps for all patients obtained with the 7 T data. From left to right, the first and second columns show the rCBV maps obtained with the spin‐echo and gradient‐echo data, respectively. The third column shows the ADC map (note the increased susceptibility artifacts when compared to the 3 T ADC maps as shown in Figure S4). The fourth column shows the so‐called Q map, where Q=∆R2*∆R23/2. Lastly, the fifth column shows the VSI maps.
**Figure S6:** Normalized distribution of vessel size estimates per patient (rows) and field strength (columns) in NAGM, showing the skewed distribution of estimates. The high values of the right‐most bins are mostly caused by outliers, as the estimates were capped at a maximum of 550 μm. To obtain the mode of the (continuous) distribution, that is, the most frequent value, kernel density estimation was performed to obtain the probability density function (black line). The mode was then obtained as the value at the maximum probability, which was 55 μm at 3 T and 44 μm at 7 T.
**Figure S7:** Normalized distribution of vessel size estimates per patient (rows) and field strength (columns) in NAWM, showing the skewed distribution of estimates. The high values of the right‐most bins are mostly caused by outliers, as the estimates were capped at a maximum of 550 μm. To obtain the mode of the (continuous) distribution, that is, the most frequent value, kernel density estimation was performed to obtain the probability density function (black line). The mode was then obtained as the value at the maximum probability, which was 56 μm at 3 T and 44 μm at 7 T.
**Figure S8:** Hysteresis loops derived from averaged GRE and SE signal in normal‐appearing gray matter (top) and white matter (bottom) for all patients. The loops are shown for 3 T (red) and 7 T (blue) and span the duration of the bolus passage. Note that the axes have a different range for patients 6 and 7.

## Data Availability

The data and analysis scripts (MATLAB) are available from the corresponding author upon reasonable request. Approval for data sharing is subject to approval by the author's local ethics committee and a formal data sharing agreement.

## Endnotes

^1^
For the sake of simplicity, we use the acronym SAGE to refer to the spin‐ and gradient‐echo implementation for both vendors. On GE‐systems, SAGE‐DSC is known as DSC‐HEPI (Hybrid EPI).
