# Supplementary material for: Feasibility of Spin‐ and Gradient‐Echo Dynamic Susceptibility Contrast MRI for Microvascular Characterization at 7 Tesla
Source: NMR Biomed. 2026 Sep 2;39(10):e70380. doi: 10.1002/nbm.70380 (PMC13538972; doi:10.1002/nbm.70380)
Supplement: Supplementary file 1 [file NBM-39-e70380-s001.docx]

**Supplementary materials**

Figure S1. A representative gradient-echo image slice and the corresponding NAGM and NAWM masks per patient (rows) and field strength (columns 1-3: 3T, columns 4-6: 7T).

***Figure S2. A representative gradient-echo image slice and the averaged NAGM signal time course for all patients for the first gradient-echo (1^st^ and 2^nd^ column) and for the spin-echo (3^rd^ and 4^th^ column) at 7T. Note the effect of the reduced GBCA dosage (patients 1-2 vs. patients 3-8) and the effect of the IV access placed in the wrist instead of the elbow (patient 5).***

***Figure S3. tSNR (left) and CNR (right) measurements at 3T (red) and 7T (blue). Results are stratified by echo (GRE on the top, SE on the bottom) and by tissue (NAGM in 1^st^ and 3^rd^ rows, NAWM in 2^nd^ and 4^th^ rows). While the tSNR was generally higher at 3T, the CNR was often higher at 7T.***

***Figure S4. Parameter maps for all patients obtained with the 3T data. From left to right, the 1^st^ and 2^nd^ column show the rCBV maps obtained with the spin-echo and gradient-echo data respectively. The 3^rd^ column shows the ADC map. The 4^th^ column shows the so-called Q map, where*** $\boldsymbol{Q=}\frac{\boldsymbol{\Delta}\boldsymbol{R}_{\boldsymbol{2}}^{\boldsymbol{*}}}{\boldsymbol{(\Delta}{\boldsymbol{R}_{\boldsymbol{2}}\boldsymbol{)}}^{\boldsymbol{3/2}}}$***. Lastly, the 5^th^ column shows the VSI maps.***

***Figure S5. Parameter maps for all patients obtained with the 7T data. From left to right, the 1^st^ and 2^nd^ column show the rCBV maps obtained with the spin-echo and gradient-echo data respectively. The 3^rd^ column shows the ADC map (note the increased susceptibility artifacts when compared to the 3T ADC maps as shown in Figure S4). The 4^th^ column shows the so-called Q map, where*** $\boldsymbol{Q}\boldsymbol{=}\frac{\boldsymbol{\Delta}\boldsymbol{R}_{\boldsymbol{2}}^{\boldsymbol{*}}}{\boldsymbol{(\Delta}{\boldsymbol{R}_{\boldsymbol{2}}\boldsymbol{)}}^{\boldsymbol{3}\boldsymbol{/}\boldsymbol{2}}}$***. Lastly, the 5^th^ column shows the VSI maps.***

*Figure S6. Normalized distribution of vessel size estimates per patient (rows) and field strength (columns) in NAGM, showing the skewed distribution of estimates. The high values of the right-most bins are mostly caused by outliers, as the estimates were capped at a maximum of 550 µm. To obtain the mode of the (continuous) distribution, i.e. the most frequent value, kernel density estimation was performed to obtain the probability density function (black line). The mode was then obtained as the value at the maximum probability, which was 55* µm at 3T and 44 µm at 7T.

***Figure S7. Normalized distribution of vessel size estimates per patient (rows) and field strength (columns) in NAWM, showing the skewed distribution of estimates. The high values of the right-most bins are mostly caused by outliers, as the estimates were capped at a maximum of 550 µm. To obtain the mode of the (continuous) distribution, i.e. the most frequent value, kernel density estimation was performed to obtain the probability density function (black line). The mode was then obtained as the value at the maximum probability, which was 56* µm at 3T and 44 µm at 7T.**

***Figure S8. Hysteresis loops derived from averaged GRE and SE signal in normal-appearing gray matter (top) and white matter (bottom) for all patients. The loops are shown for 3T (red) and 7T (blue) and span the duration of the bolus passage. Note that the axes have a different range for patient 6 and 7.***
